# Transparent Polyurethane Elastomers with Excellent Foamability and Self-Healing Property via Molecular Design and Dynamic Covalent Bond Regulation

**DOI:** 10.3390/polym17192639

**Published:** 2025-09-30

**Authors:** Rongli Zhu, Mingxi Linghu, Xueliang Liu, Liang Lei, Qi Yang, Pengjian Gong, Guangxian Li

**Affiliations:** 1 National Key Laboratory of Advanced Polymer Materials, College of Polymer Science and Engineering, Sichuan University, 24 Nanyiduan of Yihuan Road, Chengdu 610065, China; zrl19991004@163.com (R.Z.); lh10240729@163.com (M.L.); yangqi@scu.edu.cn (Q.Y.); 2 Jiangsu JITRI Advanced Polymer Materials Research Institute, Tengfei Building, 88 Jiangmiao Road, Jiangbei New District, Nanjing 211800, China; 3 Chengdu Kingfa science and technology Advanced Materials Co., Ltd., No. 665 Fujia Road, Huangjia Street, Southwest Airport Economic Development Zone, Shuangliu District, Chengdu 610213, China; liuxueliang@kingfa.com.cn (X.L.); leiliang@kingfa.com.cn (L.L.)

**Keywords:** polyurethane elastomer, supercritical CO_2_ foaming, multiple dynamic bonds, micron-pore size, self-healing

## Abstract

Microcellular thermoplastic polyurethane (TPU) foams with dynamic covalent bonds demonstrating exceptional self-healing capabilities, coupled with precisely controlled micron-scale cellular architectures, present a promising solution for developing advanced materials that simultaneously achieve damage recovery and low density. In this study, a series of self-healable materials (named as PU-S) with high light transmittance possessing two dynamic covalent bonds (oxime bond and disulfide bond) in different ratios were fabricated by the one-pot method, and then the prepared PU-S were foamed utilizing the green and efficient supercritical carbon dioxide (scCO_2_) foaming technology. The PU-S foams possess multiple dynamic covalent bonds as well as porous structures, and the effect of the dynamic covalent bonds endows the materials with excellent self-healing properties and recyclability. Owing to the tailored design of dynamic covalent bonding synergies and micron-sized porous structures, PU-S_5_ exhibits hydrophobicity (97.5° water contact angle), low temperature flexibility (*T*_g_ = −30.1 °C), high light transmission (70.6%), and light weight (density of 0.12 g/cm^3^) together with high expansion ratio (~10 folds) after scCO_2_ foaming. Furthermore, PU-S_5_ achieves damage recovery under mild thermal conditions (60 °C). Accordingly, self-healing PU-S based on multiple dynamic covalent bonds will realize a wide range of potential applications in biomedical, new energy automotive, and wearable devices.

## 1. Introduction

Maintaining the long-lasting performance of materials is one of the core demands in industrial manufacturing, resource recycling, and sustainable development [1]. It is well acknowledged that traditional polymers (such as rubber and plastic) with irreparability and non-recyclability lead to waste of resources and environmental pollution. Additionally, TPU is a type of polymeric material having a wide adjustable range and strong adaptability, which has promising application prospects in fibers, coatings, adhesives, wearable devices, biomaterials, etc. [2,3,4].

Compared with traditional TPU, the creation of self-healing TPU elastomers based on dynamic covalent bonds (DCBs) is an innovative solution due to the intelligent response and closed-loop recycling potential. The core characteristic of these materials lies in the reversible breaking and reorganization of dynamic covalent bonds, which endows the materials with self-healing capabilities, reconfigurability, and environmental adaptability. Meanwhile, combined with the supercritical carbon dioxide (scCO_2_) foaming technology, the materials can be further constructed into lightweight and highly resilient foams with multi-stage pore structures [5,6,7].

As a green physical foaming process, scCO_2_ foaming technology is non-toxic, safe, and leaves no foaming agent residue, while at the same time having the advantage of high cost-effectiveness [8,9]. It is worth noting that the introduction of dynamic covalent bonds (e.g., disulfide bond, oxime bond, hindered urea bond, etc.) allows the TPU to be triggered by thermal, optical or chemical stimuli to exchange dynamic covalent bonds when damaged, which can realize the autonomous repair of microcracks and the recovery of macroscopic mechanical and electromagnetic wave transmission properties [10]. By regulating the pressure and temperature during the scCO_2_ foaming process, the precise regulation of the cell size can be realized without foaming agents, thereby effectively optimizing the microstructure of materials and obtaining a closed-cell foam system with better uniformity of the cell distribution and controllable geometrical parameters [11,12,13,14].

In consequence, the collaborative design of the two structures not only breaks through the limitation of “one-time use” of traditional foams but also realizes the in situ repair and recycling regeneration of materials [15,16,17,18]. For example, embedding dynamic covalent bonds into the molecular chains of TPU elastomers can provide self-repairing performance and recyclability, which not only reduces the maintenance cost but also prolongs the service life of the products [19]. Zhou et al. [20] prepared double-locked covalent adaptive networks (DLCANs) materials with both ultra-stable crosslinked structures and programmed degradation properties, which possess UV shielding, self-repairing, and controllable peeling properties. Liu et al. [21] successfully prepared a high-strength self-healing PU elastomer based on the synergistic effect of dynamic oxime–carbamate bonds of the main chains and thiocarbamate bonds of the crosslinking sites, and the self-repair efficiency of the elastomer reaches 95.5% after repair under mild conditions; the recovery rate of its mechanical properties is more than 100% after multiple thermal compression cycles. Nonetheless, this kind of material with a tightly packed three-dimensional covalent structure will seriously restrict the free movement of molecular chains, resulting in a reduction in the elongation at break of the material and making it difficult to meet the requirements of foaming materials for ultra-high flexibility and homogeneity of cells [22,23].

Based on the design concept of constructing materials with both excellent self-healing performance and stable foam structure, Zheng et al. [24] synthesized a polydimethylsiloxane elastomer with a dual-network structure, which exhibits an excellent self-healing efficiency of 93%, along with good tensile strength and elongation ratio. In addition, the self-healing elastomeric foam was prepared using scCO_2_ foaming technology, which could achieve microcrack repair at 80 °C and maintain a stable cell morphology. Patrick et al. [25] developed a self-repairing polymer foam material based on microvascular networks encapsulating the two-component PU repair agent, with >100% fracture toughness recovery, and achieved rapid repair in minutes at room temperature and efficient recovery from multiple damage cycles. Rashid et al. [26] developed a lightweight foam aggregate by geopolymerization and microwave curing and achieved multifunctional integration of excitation foaming repair with sodium silicate. Furthermore, the experimental results showed that the material achieved 100% autonomous repair efficiency and possessed significant recovery of compressive/flexural strength, providing a new multifunctional synergistic strategy for self-repairing concrete [27,28,29,30]. It can be seen that, based on the multifunctional synergistic design strategy to build a material system with both a dynamic self-healing mechanism and stability of the cell structure, not only can it achieve high self-healing efficiency and multiple-cycle repair capability through the dynamic covalent bond structure, but also can achieve lightweight with scCO_2_ foaming technology [31,32,33].

Given this, a dynamic TPU with excellent foaming stability and self-healing ability under mild conditions was constructed by introducing oxime bond (-C=N-) and dynamic disulfide bond (-S-S-) into TPU molecular chains by reaction. In this study, a unique strategy is proposed to simultaneously enhance the self-healing and foaming properties of TPU. Specifically, self-healing polyurethane (PU-S) containing multiple dynamic bonds (oxime, disulfide, and urethane bonds) was first synthesized from polycarbonate diol (PCDL) with a molecular weight of 2000 g/mol, isophorone diisocyanate (IPDI), dimethylglyoxime (DMG), and 2,2′-Dithiodiethanol (HEDS). Subsequently, a series of self-healing PU-S foams containing multiple dynamic bonds was prepared via scCO_2_ foaming technology at different foaming conditions. It is noteworthy that the PU-S films and foams containing multiple dynamic covalent bonds both exhibit excellent self-healing properties at 60 °C after destructive damage. In addition, the effect of multiple dynamic covalent bonds endows materials with recyclability, and materials can be molded by repeated hot pressing with little effect on their properties. The multiple dynamic covalent structures of the PU-S films and the corresponding influence on their scCO_2_ foaming were systematically investigated by studying the thermal, mechanical, and self-healing properties, as well as the micro-morphological characteristics. This study not only lays a key theoretical foundation and technical path for the extension of the service life of self-healing materials and their foams, in addition to the construction of a green and sustainable material system, but also provides promising material solutions for the fields of intelligent robotics, biomedical, new energy vehicles, intelligent stealth and electromagnetic protection, flexible electronics, and wearable devices.

## 2. Materials and Methods

### 2.1. Materials

Polycarbonate diol (PCDL, *M_n_* = ~2000 g/mol), dibutyltin dilaurate (DBTDL, 95%), and 2,2′-Dithiodiethanol (HEDS, 90%) were purchased from Shanghai Aladdin (Shanghai, China). Dimethylglyoxime (DMG, 90%) was obtained from Hangzhou Yanqu Information Technology Co., Ltd. (Hangzhou, China). Isophorone diisocyanate (IPDI, 90%) was purchased from Sigma-Aldrich Trading Co., Ltd. (Shanghai, China). N, N-Dimethylformamide (DMF, 90%) was purchased from Shanghai Maclean Biochemical Technology Co., Ltd. (Shanghai, China). Additionally, PCDL were dried at 120 °C under vacuum (−0.1 MPa) for 2 h to remove water and impurities, and other chemicals were used without any further purification [34,35].

### 2.2. Methods of Synthesis

#### 2.2.1. Synthesis of PU-S Elastomers

**Figure 1:**
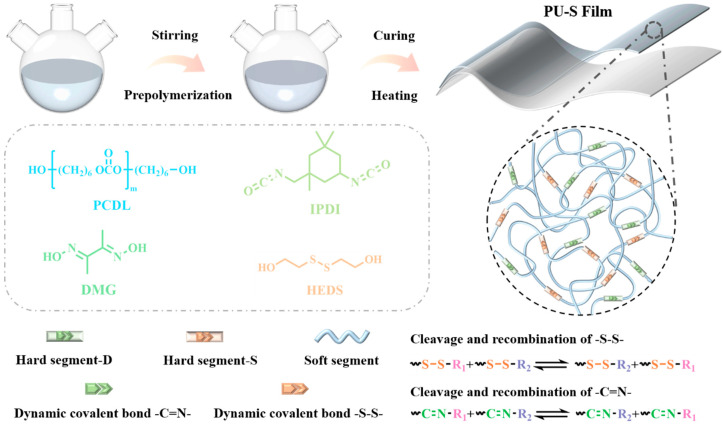
Synthetic route of samples.

As an example of the synthesis procedure of PU-S_5_, IPDI (10.5 g, 47.0 mmol), purified PCDL (13.0 g, 6.5 mmol), catalyst DBTDL (10 μL), and DMF (15 mL) were first added into a three-necked flask equipped with a mechanical stirrer and a nitrogen inlet, as shown in Figure 1. The three-necked flask filled with the reaction ingredients was then placed in an oil bath at a temperature of 100 °C, and the reaction was carried out for 2 h at a constant temperature in a stream of nitrogen with a stirring rate of 150 r/min. Then, the temperature of the oil bath was lowered to 80 °C, and DMG (1.4 g, 12.0 mmol) and HEDS (1.8 g, 12.0 mmol) were added sequentially for 1 h to obtain PU-S_5_ elastomer [36]. After the reaction, the PU-S_5_ elastomer was poured onto a tetrafluoro plate and cooled at room temperature for 12 h. The samples were then put into a blast oven for solvent removal, and the temperature range of the oven was set from 80 °C to 120 °C, which was increased by 10 °C at intervals of 1 h, to obtain the transparent PU-S_5_ films. The specific calculation method includes fixing the ratio of the total mass of the hard segments (IPDI, HEDS, and DMG) to the total weight of the reactants at 40%. Fixing the *R* value (the molar ratio of isocyanate groups (-NCO) to hydroxyl groups (-OH)) at 1.4. Then, according to the different molar ratios of HEDS and DMG, the samples with molar ratios of 0:10, 2:8, 5:5, 8:2, and 10:0 were named as PU-S_0_, PU-S_2_, PU-S_5_, PU-S_8_, and PU-S_10_, respectively.

#### 2.2.2. Preparation of PU-S Foams

The synthesized PU-S films were cut into pieces and then put into customized stainless-steel molds. The top and bottom of the molds were wrapped with PTFE films and then put into hot presses with a set pressure of 10 MPa, a temperature of 130 °C, and the hot press was carried out for 10 min to obtain flakes with fixed shapes and sizes. Then, the whole foaming process includes three steps: pressurization, infiltration, and pressure relief [37]. Firstly, the flakes were put into a small hollow cage made of iron wire to ensure that the flakes could be uniformly exposed to CO_2_, and then the small cage was put into the autoclave, and the lid was tightened. When the temperature and pressure reached the set values, CO_2_ was in a supercritical state. Further, the inlet and outlet valves of the autoclave were closed, the autoclave was kept airtight, and the flakes were moistened for 2 h so that the CO_2_ was fully diffused therein, and finally, the lid of the autoclave was opened after rapid pressure relief to obtain the PU-S foams.

### 2.3. Characterization

Fourier transform infrared spectroscopy (FT-IR): (1) A Nicolet 6700 FT-IR spectrometer manufactured by Thermo Fisher Scientific (Waltham, MA, USA) was used for the structural analysis of PU-S in total reflection mode. The spectral resolution was 0.4 cm^−1^, the number of scans was 32 times, the wave number accuracy was 0.01 cm^−1^, and the scanning range was 400~4000 cm^−1^; (2) two-dimensional correlation infrared spectroscopy, the test temperature range was 15~200 °C, the scanning range was 400~4000 cm^−1^, and the data of the elevated temperature infrared spectroscopy were recorded at intervals of 5 °C, and the accuracy of the temperature control was 0.1 °C. Based on the measured warming infrared spectral data, the 2D infrared spectra were calculated and plotted using the 2D infrared software (2DCS professional version) [38].

Nuclear magnetic resonance hydrogen spectroscopy (^1^H-NMR): the structures of the prepared PU-S were further characterized and analyzed using an AV III HD 400 MHz NMR instrument from Bruker (Karlsruhe, Germany). And 1.0 mg of the prepared samples was dissolved in deuterated chloroform, and tetramethylsilane (TMS) was used as an internal standard for structure analysis [39].

Ultraviolet-visible spectrophotometer (UV–Vis): The UV-3600 UV–Vis spectrophotometer manufactured by Shimadzu Corporation (Kyoto, Japan), was used to test the PU-S films in the transmittance mode, with the thickness of the film samples being 1.0 mm, and the scanning rate of the scanning rate being medium, with a scanning range of 200~800 nm [40].

Mechanical properties: The tensile properties of PU-S films were evaluated using an Instron 5567 universal testing machine manufactured by Instron Corporation (Norwood, MA, USA) under room temperature conditions. The films, with a thickness of approximately 1 mm, were subjected to a crosshead speed of 100 mm/min during testing [41]. The healing efficiency (*η*) of the samples was calculated using Equation (1), where *σ_original_* and *σ_healed_* are the tensile strengths of original and healed samples:

(1) $$\eta =\;\frac{\sigma_{healed}}{\sigma_{original}}100\%\;$$

Scanning electron microscope (SEM): (1) The microscopic morphology of the cells in the cross-section of PU-S foams was observed using a JSM-606 scanning electron microscope produced by Electron Optics Laboratory Corporation (Japan), and all the samples were glued to the sample stage with conductive adhesive tapes for the spraying of gold before being observed. (2) The self-repairing PU-S_0_ film, PU-S_5_ film, and PU-S_5_ foam were cut off with a scalpel, and then the two cut-off parts were contacted and put into an oven at 60 °C for a self-repairing process for 5 h. A scanning electron microscope was used to observe the morphology of the cut-off places before and after the self-repairing of the samples [42].

Expansion ratio and void fraction: The density (*ρ*_solid_ and *ρ*_foam_) was calculated from the mass and volume of the unfoamed and foamed samples, respectively, and the expansion ratio (*ER*) of the foamed samples was calculated using Equation (2):

(2) $$ER=\;\frac{\rho solid}{\rho foam}\;$$

*ER* was brought into Equation (3) to obtain void fraction (*VF*):

(3) $$VF\;=\;\frac{\left(ER-1\right)}{ER}\;\times \;100\%$$

Average cell size and cell density: The average cell size (*D_n_*) of the foamed samples was calculated using Equation (4), where *n_i_* is the number of regions with a diameter of *D_i_* quantified from the SEM images using ImageJ software (version: 1.8.0):

(4) $$D_{n}=\;\frac{\sum_{i}\left(n_{i}D_{i}\right)}{\sum_{i}n_{i}}\;$$

Cell density (*N_f_*) was calculated using Equation (5), where *n* is the number of cells within a specific region (*S_A_*) in the SEM images:

(5) $$N_{f}=\;{\left(\frac{n}{S_{A}}\right)}^{1.5}\;\times \;ER$$

## 3. Results and Discussion

### 3.1. Functional Groups of Synthesized PU Films

**Figure 2:**
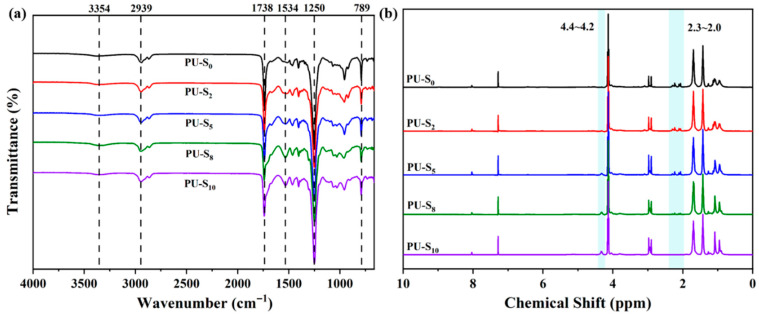
(**a**) FT-IR spectra and (**b**) ^1^H NMR spectra (in CDCl_3_, @400 MHz) of PU-S_0_, PU-S_2_, PU-S_5_, PU-S_8_, and PU-S_10_.

As shown in Figure 2a, the FTIR analysis revealed characteristic absorption bands at 3354 cm^−1^ and 1534 cm^−1^, which were assigned to the stretching vibration of N-H bonds and in-plane bending vibration of the urethane group (-NHCOO-) in the polymer matrix, respectively. The absorption peaks observed at 2939 cm^−1^ and 789 cm^−1^ corresponded to the stretching and bending vibrations of C-H bonds. Additionally, the stretching vibrations of C=O and C-O-C groups were identified at 1738 cm^−1^ and 1250 cm^−1^, respectively. Notably, the complete absence of characteristic absorption at 2260 cm^−1^ (-NCO stretching) confirmed the full consumption of isocyanate groups from IPDI precursor through their reaction with hydroxyl groups in PCDL, DMG, and HEDS. These spectroscopic findings collectively demonstrate the successful formation of urethane linkages and the synthesis of the target compound [43]. The NMR analysis revealed that the characteristic peaks at chemical shifts of 4.4~4.2 ppm and 2.3~2.0 ppm correspond to the methylene protons adjacent to disulfide bonds and methyl protons adjacent to oxime linkages in PU-S, respectively, as shown in Figure 2b. The remaining chemical shifts showed good agreement with those documented in the literature [44]. In addition, as shown in Figure S1, it also provides direct evidence for the successful bonding of the target product. Furthermore, the gel permeation chromatography (GPC) analysis was performed on the synthesized polymers prior to the thermal treatment (80~120 °C) to characterize their initial molecular weight distributions. The macromolecular properties of the samples, including the number-average molecular weight (*M_n_*), weight-average molecular weight (*M_w_*), and polydispersity index (*Đ*) were shown in Figure S2 and Table S1. Notably, the results indicate that the *Đ* exhibits a gradually increasing trend with higher DMG content. This is attributed to the increased proportion of oxime–carbamate bonds within the molecular chains. The dynamic exchange reactions of these bonds facilitate the scission and recombination of polymer chains during the polymerization process, thereby enhancing the polydispersity of the molecular weight distribution. Additionally, a higher DMG content may also indirectly affect the freedom of chain mobility by influencing the packing density or phase separation behavior in the hard segment regions, further contributing to the broadening of the molecular weight distribution.

**Figure 3:**
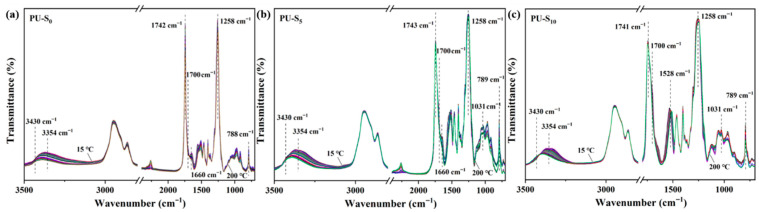
In situ FT-IR spectra of (**a**) PU-S_0_; (**b**) PU-S_5_; (**c**) PU-S_10_ at 15~200 °C.

In order to investigate the effect of dynamic oxime–ammonia and disulfide bonds at high temperatures, the elevated temperature infrared spectra of PU-S_0_, PU-S_5_, and PU-S_10_ were investigated at 15~200 °C, respectively. As shown in the Figure 3a–c, a decrease in the intensity of the absorption peaks at 3354 cm^−1^ and 1700 cm^−1^ was observed in the elevated temperature infrared spectra of all the three PU-S samples, revealing the hydrogen bond breakage between the N-H and C=O groups in the hard segments; for PU-S_0_ and PU-S_5_, the dynamic oxime–ammonia ester bonding at 1660 cm^−1^ was observed in their infrared spectra when the temperature was 15 °C. The absorption peaks of the dynamic oxime–ammonia ester bonds were gradually weakened or even disappeared when the temperature was increased to 200 °C, indicating that the dynamic oxime–ammonia ester bonds in PU-S_5_ and PU-S_0_ were gradually dissociated; for PU-S_5_ and PU-S_10_, a gradual decrease in the intensity of the absorption peak of the dynamic disulfide bonds near 1031 cm^−1^ was observed on their infrared spectra, which revealed the successful introduction of the dynamic covalent bonds [45].

**Figure 4:**
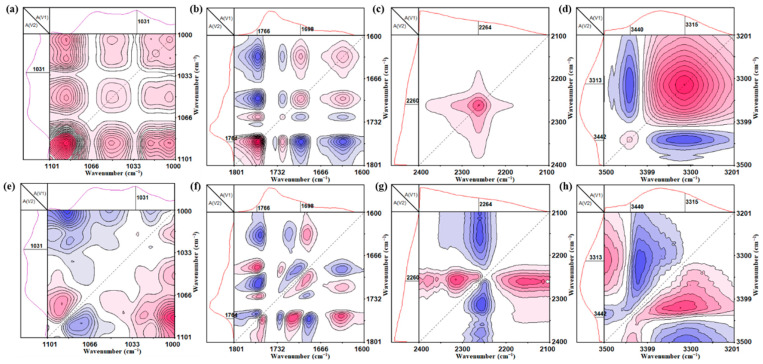
Synchronous 2D correlation maps of PU-S_5_ at (**a**) 1000~1101 cm^−1^, (**b**) 1600~1801 cm^−1^, (**c**) 2100~2400 cm^−1^, and (**d**) 3201~3500 cm^−1^ and asynchronous 2D correlation maps of PU-S_5_ at (**e**) 1000~1101 cm^−1^, (**f**) 1600~1801 cm^−1^, (**g**) 2100~2400 cm^−1^, and (**h**) 3201~3500 cm^−1^ monitored during the total observation period at 15~200 °C.

Figure 4a–h show the two-dimension (2D) correlation synchronous and asynchronous spectra of PU-S_5_ at 1000~1101 cm^−1^, 1600~1801 cm^−1^, 2100~2400 cm^−1^, and 3201~3500 cm^−1^, respectively. The positive auto-peaks at 3300 cm^−1^ and 3400 cm^−1^ in the synchronous spectrum reveal the temperature-dependent intensity variations, while their cross-peaks indicate opposite directional changes, reflecting the reorganization of the hydrogen-bonding network around -NH groups. This reorganization is likely driven by the dynamic covalent bond exchange processes [46]. Critically, the positive auto-peak at 2260 cm^−1^ in the synchronous spectrum demonstrates a temperature-induced intensity increase, providing direct evidence for the generation of free-NCO groups. This is a key spectroscopic signature of the reversible cleavage of the oxime–urethane bonds, confirming the active exchange dynamics within the system [47]. Synchronous spectral analysis of the 1760 cm^−1^ and 1700 cm^−1^ regions shows positive auto-peaks corresponding to temperature-dependent variations in free carbonyl C=O and hydrogen-bonded carbonyl C=O groups, respectively. Their cross-peaks with opposite phase directions evidence the dynamic equilibrium between free and hydrogen-bonded carbonyl states, a process that is intimately coupled to the mobility granted by covalent bond exchange. Furthermore, the positive auto-peak at 1031 cm^−1^ in the synchronous spectrum reveals temperature-responsive intensity changes, indicating the molecular rearrangement and metathesis reactions of disulfide bonds [48]. Collectively, the 2D correlation analysis not only captures the temperature-dependent changes in hydrogen bonding but, more importantly, pinpoints the spectral evidence of the underlying dynamic covalent bond exchange (both oxime bond and disulfide bond) that enables the macroscopic self-healing behavior.

### 3.2. Optical and Mechanical Properties of Synthesized PU Films

**Figure 5:**
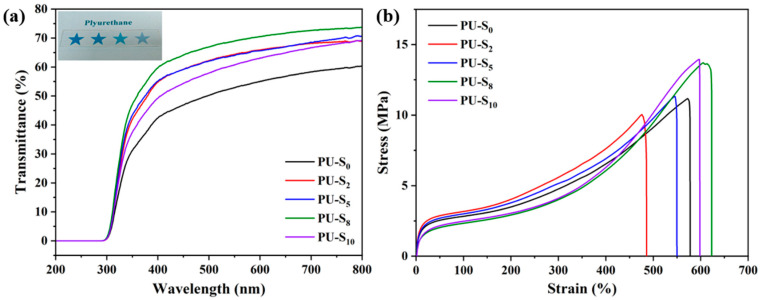
(**a**) Transmittance spectrum and optical photograph, (**b**) stress–strain curves of PU-S_0_, PU-S_2_, PU-S_5_, PU-S_8_, and PU-S_10_ at a thickness of 1.0 mm.

As shown in the optical photograph of PU-S_8_, it is colorless and transparent, and the colorful pentagram pattern underneath is visible, as shown in Figure 5a. The transparency of different samples in the visible light region was then tested by using a UV–Vis. At room temperature, the transmittance of the samples ranged from 60.4% (PU-S_0_) to 73.9% (PU-S_8_) within the wavelength range of 320~800 nm, as shown in Figure 5a. Furthermore, the transmittance of the samples exhibits an increasing trend with the introduction of HEDS, corresponding to a decrease in the content of -C=N- groups and an increase in the content of -S-S- groups within the molecular chains. This is because the strong polarity, hydrogen-bonding capability, and rigidity of the -C=N- group exacerbate microphase separation and can also lead to the formation of conjugated structures. These effects result in larger and more heterogeneous phase domain sizes, thereby reducing transmittance. Conversely, the -S-S- group, owing to its weak polarity, minimal hydrogen-bonding ability, and excellent flexibility, promotes the formation of a finer and more uniform nanoscale microphase-separated structure. The size of these domains is significantly smaller than the wavelength of light, which drastically reduces light scattering and consequently enhances transmittance [49]. Additionally, the X-ray diffraction (XRD) spectra of PU-S samples with different DMG and HEDS contents are shown in Figure S4. The results indicate that the molecular structures of all samples exhibit an amorphous state and belong to random polymers, which is consistent with the transmittance test results. Furthermore, Figure S3 also demonstrates that the samples exhibit good hydrophobicity.

As shown in Figure 5b, the mechanical test results indicate that PU-S_0_, PU-S_2_, PU-S_5_, PU-S_8_ and PU-S_10_ exhibit different tensile strengths of 11.2 ± 0.5 MPa, 10.0 ± 0.5 MPa, 11.3 ± 0.5 MPa, 13.7 ± 0.5 MPa, and 14.0 ± 0.5 MPa; the elongation at break was 586.6%, 495.2%, 558.6%, 656.2%, and 611.0%, respectively (data summarized in Table S4). The above results indicate that as the HEDS content increases, the tensile strength of the samples exhibits a slight improvement. This is due to the dynamic crosslinking compensating for the loss of polarity resulting from the reduction in -C=N- groups, while simultaneously optimizing the phase-separated structure. Notably, the elongation at break of the samples shows a significant enhancement with increasing HEDS content. This pronounced improvement stems from the increased flexibility of the molecular chains and the energy dissipation mechanism of the dynamic bonds, which facilitate easier sliding and reorganization of the polymer chains [50]. In addition, the dynamic mechanical properties of the samples were characterized by dynamic mechanical analysis (DMA), and the results are shown in Figure S6. The results indicate that PU-S_10_ exhibits higher damping properties over a wider temperature range. These test results are also consistent with those obtained from differential scanning calorimetry (DSC) (Figure S5 and Table S2). Moreover, the thermal gravimetric analysis (TGA) results presented in Figure S7 and Table S3 demonstrate that the samples possess good thermal stability.

### 3.3. Foaming Behavior of Synthesized PU Films

**Figure 6:**
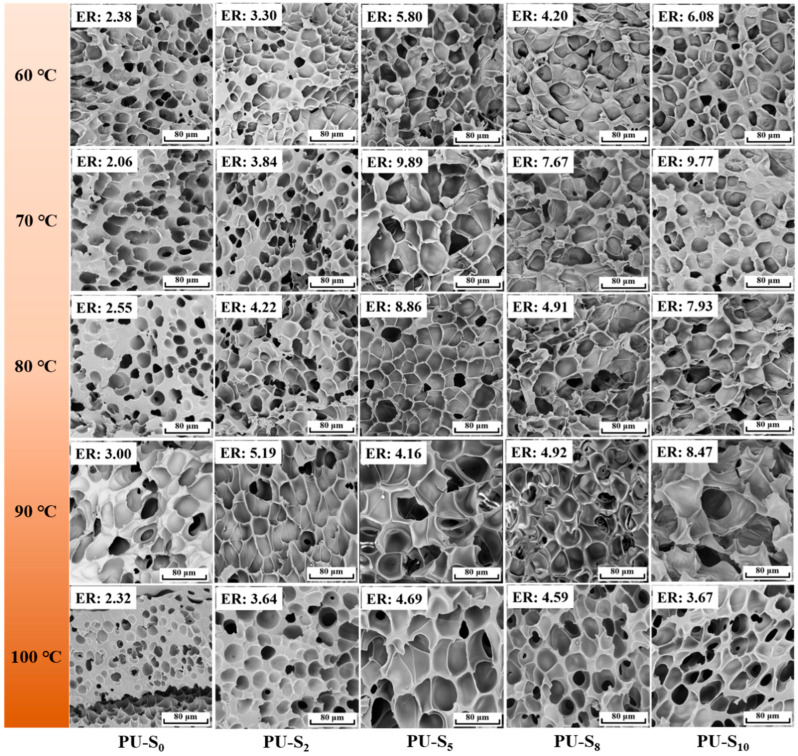
SEM images of foam samples at various foaming temperatures.

**Figure 7:**
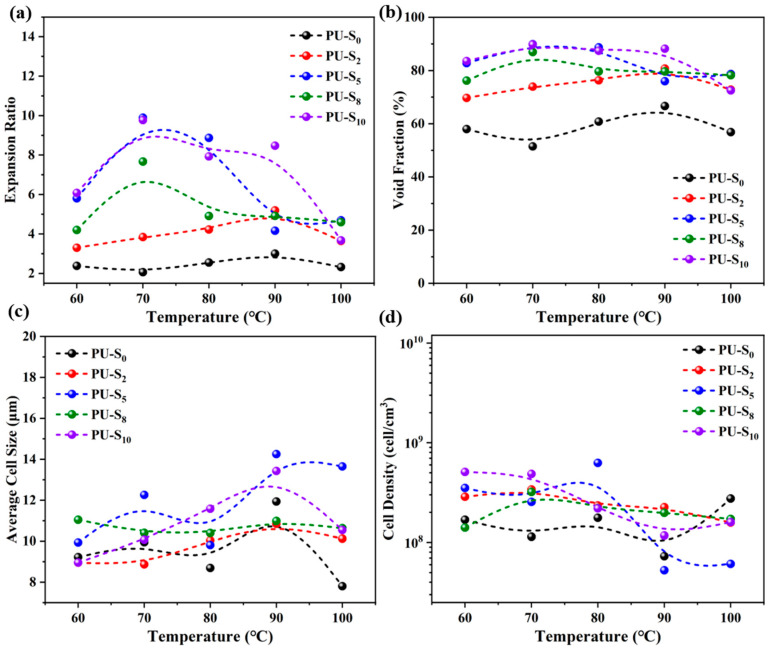
(**a**) Expansion ratio, (**b**) void fraction, (**c**) cell size, and (**d**) cell density of foam samples at various foaming temperatures.

SEM was used to observe the cross-section of the PU-S foams for the study. Figure 6 shows the morphology of cells in foams PU-S_0_, PU-S_2_, PU-S_5_, PU-S_8_, and PU-S_10_ at different foaming temperatures. Additionally, Figure 7a–d shows the expansion ratios, void fraction, cell size, and cell density of samples at various foaming temperatures, respectively. When the HEDS/DMG molar ratio was 5:5, the cell size of PU-S_5_ increased slightly (the average cell size was 14.3 μm [Figure 7c]), the cell density was 2.6 × 10^8^ cell/cm^3^ [Figure 7d], and the expansion ratio increased significantly (about 10 folds [Figure 7a]) with the highest void fraction (90% [Figure 7b]) at a saturation pressure of 15 MPa, a foaming temperature of 70 °C, and a foaming time of 1 h. With the increase in HEDS content, the expansion ratio of foams containing dynamic covalent bonds increased and then decreased at different foaming temperatures. This suggests that the foaming process, as well as the morphology of the bubbles in the foam samples, may be significantly affected due to the differences in the molecular chain structure as well as the HEDS and DMG contents. Due to the synergistic regulation of melt strength at all stages of foaming by disulfide bonding and imine bonding with the increase in HEDS content, as well as the synergistic hydrogen bonding-dynamic bonding to reduce the melt viscosity while maintaining the stability of the bubble wall, the cell size and expansion ratios of the PU foams increased [51]. However, further increasing the HEDS content causes the molecular system to lose its rigidity. This excessive fluidity from the dynamic bonds causes the melt strength of PU-S to collapse, preventing stable cell growth [52].

### 3.4. Self-Healing Performance of Synthesized PU Films and the Corresponding Foams

**Figure 8:**
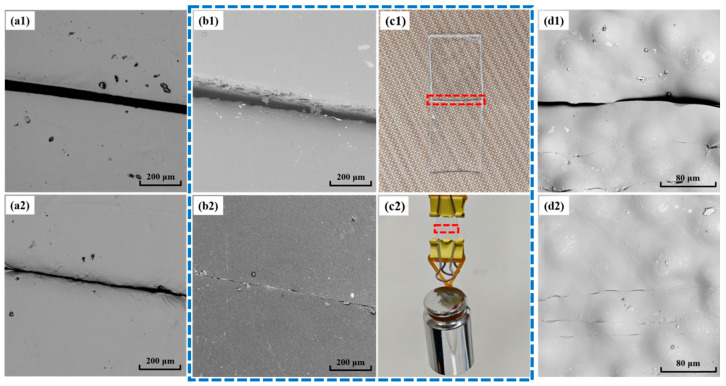
SEM image of the cutting position of (**a1**) PU-S_0_ film, (**b1**) PU-S_5_ film, and (**d1**) PU-S_5_ foam before heat treatment; (**a2**) PU-S_0_ film, (**b2**) PU-S_5_ film, and (**d2**) PU-S_5_ foam after heat treatment; (**c1**) photograph of PU-S_5_ film before self-healing; (**c2**) photograph of PU-S_5_ film after self-healing.

To assess the self-healing ability of PU-S, the PU-S film was cut in half using a surgical scalpel, and then the fracture interfaces were aligned to ensure maximum surface contact. The treated samples were placed in an oven at 60 °C, and the healing of the scratches in the same part of the samples was observed after 5 h using SEM. Figure 8a1, b1, and d1 show the crack morphology of the PU-S_0_ film, PU-S_5_ film, and PU-S_5_ foam, respectively, prior to heat treatment. Corresponding SEM images of the cut locations after heat-induced healing for the PU-S_0_ film, PU-S_5_ film, and PU-S_5_ foam are presented in Figure 8a2, b2, and d2, respectively. Figure 8c1 and c2 show a photograph of PU-S_5_ film before self-healing and the healed PU-S_5_ film under tension from a suspended 100 g weight, respectively. Additionally, the stress–strain curves of the samples after self-healing are shown in Figure S8, and their corresponding tensile strengths and *η* are listed in Table S4. The results demonstrate that the cracks in both the PU-S_5_ film and foam significantly narrowed after self-healing, with the cut traces virtually disappearing. In contrast, the cut marks remained visible in the PU-S_0_ film. This disparity is attributed to the absence of -S-S- structures within the molecular chains of PU-S_0_, which results in a deficiency of low-temperature dynamic bonding assistance, consequently leading to its inferior self-healing performance. Furthermore, the results indicate that as the relative content of -S-S- in the polymer increases, the *η* of the material gradually improves. This is primarily attributed to the strong ability of disulfide bonds to undergo symmetric exchange reactions under thermal stimulation, whereby their dynamic reversibility facilitates the reconnection of molecular chains and the rearrangement of topological structures at the damaged interface. Meanwhile, the oxime group also exhibits dynamic characteristics (such as reversible oxime–urethane exchange), but its bond energy and dissociation kinetics generally differ from those of disulfide bonds. When the proportion of disulfide bonds is increased, the overall activation energy for bond exchange within the system decreases, and the mobility of polymer segments is enhanced, thereby enabling efficient self-healing even under lower temperature stimuli. However, an excessively high content of disulfide bonds may compromise the mechanical integrity after healing. Therefore, an optimized ratio between the oxime group and disulfide bonds is required to strike a balance between dynamic reactivity and system stability, thereby achieving both excellent self-healing performance and mechanical strength.

## 4. Conclusions

In this study, self-repairable micron-sized porous PU-S foams were prepared by stepwise polymerization, nucleophilic addition reaction, and scCO_2_ foaming technology. Moreover, the introduction of HEDS (with disulfide bonding) and DMG (with oxime bonding) can modulate the dynamic covalent bonding structure of PU-S, which can endow self-repairing effects to the PU-S films and foams. The addition of HEDS to PU-S increases the number of -S-S- bonds in the molecular chain of the material, which is conducive to the enhancement of dynamic exchange and reaction efficiencies of the material under mild conditions. The presence of -C=N- bonds in DMG increases the concentration of polar functional groups and enhances hydrogen bonding density. Conversely, the incorporation of HEDS imparts greater flexibility to the molecular chains, thereby improving the material’s elongation at break. At a HEDS/DMG molar ratio of 5:5, PU-S_5_ exhibits the best foaming performance (with a foaming ratio approaching 10-fold), high light transmittance (70.6%). Thanks to the tailored dynamic covalent bonding synergies and micron structure design, PU-S films and foams exhibit excellent self-healing properties under mild conditions (60 °C) after destructive damage.

## Data Availability

The original contributions presented in this study are included in the article/Supplementary Material. Further inquiries can be directed to the corresponding authors.

## Supplementary Materials

The following supporting information can be downloaded at: https://www.mdpi.com/article/10.3390/polym17192639/s1, Figure S1: (a) XPS spectra of PU-S_5_; (b) high-resolution spectrum of PU-S_5_, including C 1s (b1), O 1s (b2), N 1s (b3), and S 2p (b4); (c) Reconfigurability of PU-S_5_; Figure S2: GPC curves of samples; Figure S3: Water contact angle measurement of samples; Figure S4: XRD spectra of samples; Figure S5: DSC curves of samples in the temperature range from –60 °C to 100 °C; Figure S6: Tan δ curves of samples; Figure S7: (a) TG curves of the samples; (b) DTG curves of the samples; Figure S8: Stress-strain curves of the healed samples; Table S1: Macromolecular characteristics of samples; Table S2: DSC data of samples; Table S3: The TGA data of the samples [53,54,55,56,57,58,59,60,61,62,63].
