# Supplementary material for: Transparent Polyurethane Elastomers with Excellent Foamability and Self-Healing Property via Molecular Design and Dynamic Covalent Bond Regulation
Source: Polymers (Basel). 2025 Sep 30;17(19):2639. doi: 10.3390/polym17192639 (PMC12526580; doi:10.3390/polym17192639)
Supplement: Supplementary file 1 [file polymers-17-02639-s001.zip › Supplementary.pdf]

Supplementary Information

Characterization

X-ray photoelectron spectroscopy (XPS): The prepared PU-S were tested using a K-Alpha X-ray photoelectron spectrometer manufactured by Thermo Scientific (Waltham, MA, USA) operated at 12 kV and 6 mA filament current.

Gel permeation chromatography (GPC): The molecular weights and molecular weight distributions of PU-S were determined using an HLC-8320GPC gel permeation chromatograph manufactured by Tosoh Corporation (Tokyo, Japan). The testing conditions were room temperature, and the mobile phase was DMF at a flow rate of 0.4 mL/min.

Water contact angle (WCA): The water contact angles of PU-S films were tested using a SINDIN SDC-350 contact angle tester manufactured by Suzhou Fubaite Instrument Technology Corporation (Suzhou, China), under the conditions of room temperature and distilled water as the test liquid.

X-ray diffraction (XRD): PU-S were characterized using an Ultima IV X-ray diffractometer manufactured by Rigaku Corporation (Tokyo, Japan), under the test conditions of room temperature, Cu target, tube voltage of 20 ~ 60 KV, 2θ range of 10° ~ 60°, and goniometer radius of 185 mm.

Differential scanning calorimetry (DSC): The Q250 Differential Scanning Calorimeter produced by TA Instruments (New Castle, DE, USA), was used to conduct the test in a nitrogen atmosphere, and the test mode was "heating up-cooling down-heating up" to eliminate the thermal history of the material, with the temperature range of –60 ℃ ~ 100 ℃, and the rate of heating up was 10 ℃/min.

Dynamic mechanical analysis (DMA): The dynamic mechanical properties of PU-S were tested using the Q850 Dynamic Thermo-mechanical Analyzer manufactured by TA Instruments (New Castle, DE, USA). The temperature scanning was carried out in tensile mode with a test frequency of 1.0 Hz, an amplitude of 15.0 μm, a temperature increase rate of 5.0 ℃/min, and a test temperature range of –80 ℃ ~ 180 ℃.

Thermal gravimetric analysis (TGA): The thermal stability of PU-S was evaluated using a NETZSCH TG209 thermogravimetric analyzer (Selb, Bavaria, Germany) under nitrogen atmosphere with a heating rate of 10 ℃/min over a temperature range of 50 ℃ ~ 700 ℃.

Results and discussion

As shown in Figure S1 (a), the XPS analysis demonstrated excellent agreement between the electron binding energies of C, N, O, and S in PU-S_5 and those reported in the literature [1]. It is worth noting that in Figure S1 (b1), the peaks in C 1s XPS spectrum at 284.80 eV, 286.79 eV and 289.25 eV assigned to the -C-C-/-C-H, -C-O-C-/-C-N- and -C=O of PU-S_5 were observed, respectively. Figure S1 (b2) also shows the deconvolution of O 1s, mainly existing in two forms: -C=O (532.25 eV) and -C-O-C- (533.83 eV). Furthermore, the N 1s spectrum in Figure S1 (b3) exhibits a deconvolution peak at 399.87 eV, corresponding to the -NHCO group in PU-S_5. Significantly, the S 2p XPS spectrum of PU-S_5 exhibits two peaks at 163.94 eV and 169.03 eV, corresponding to -C-S- and -S-S-, which provides direct evidence for the successful binding of the target product [Figure S1 (b4)]. In addition, as shown in Figure S1 (c), due to the synergistic effect of multiple dynamic covalent bonds, PU-S_5 has excellent recyclability [2].

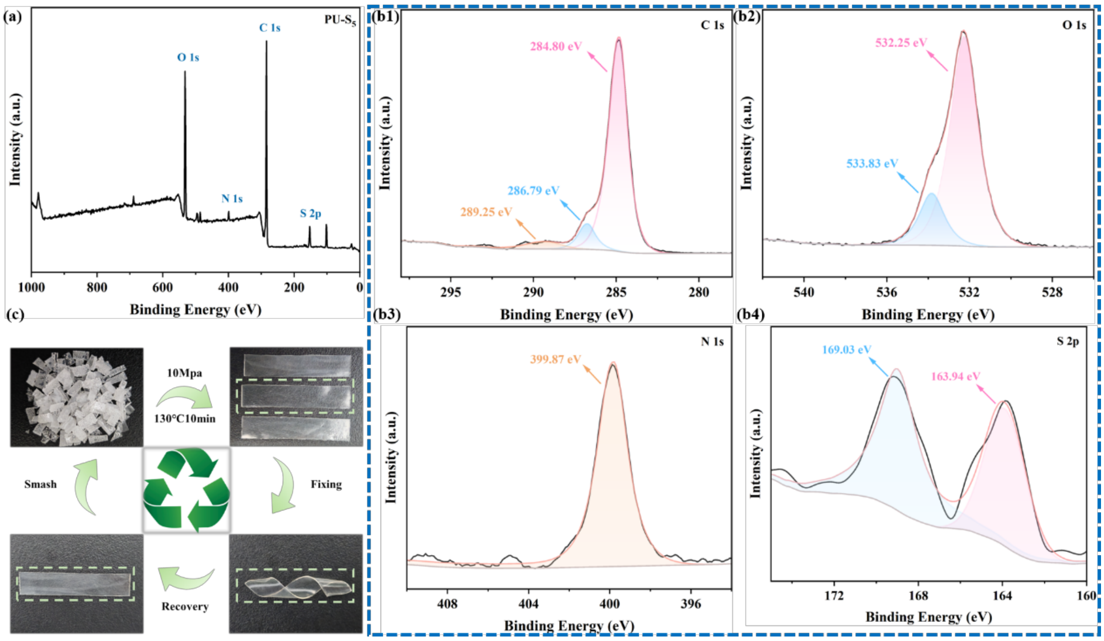

Figure S1. (**a**) XPS spectra of PU-S_5; (**b**) high-resolution spectrum of PU-S_5, including C 1s (**b1**), O 1s (**b2**), N 1s (**b3**), and S 2p (**b4**); (**c**) Reconfigurability of PU-S_5.

The GPC curves of the samples are shown in Figure S2, and the molecular characteristics are summarized in Table S1. The GPC analysis revealed that the synthesized samples exhibited weight-average molecular weight (M_w) in the range of 17,218 ~ 46,192 g/mol, with polydispersity index (Đ) spanning 1.7 ~ 3.4 for all samples.

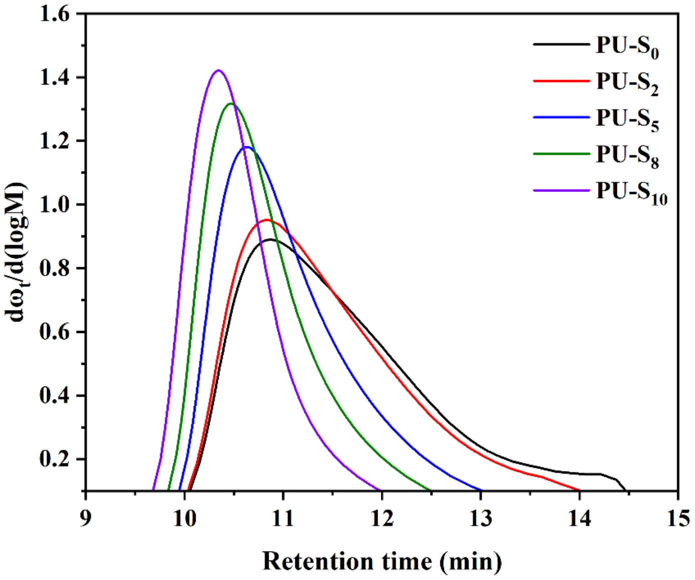

Figure S2. GPC curves of samples.

Table S1. Macromolecular characteristics of samples

**Samples**	**Molar ratio (HEDS: DMG)**	M_n **(g/mol)**	M_w **(g/mol)**	**Đ**
PU-S_0	0:10	5041	17,218	3.4
PU-S_2	2:8	6038	18,707	3.1
PU-S_5	5:5	11,244	25,810	2.3
PU-S_8	8:2	17,821	33,692	1.9
PU-S_{10}	10:0	27,120	46,192	1.7

M_n refers to number-average molecular weight.

M_w refers to weight-average molecular weight.

Đ refers to polydispersity index.

Water contact angle data for all samples with varying DMG and HEDS contents are presented in Figure S3. As shown in the figure, sample PU-S_0 exhibits a water contact angle of 74.0°. Following the introduction of HEDS, the PU-S samples demonstrate an increasing trend in water contact angles, indicating a gradual transition toward hydrophobic surface properties. Notably, PU-S_5 achieves the highest contact angle of 97.5°, representing a 23.5° increase compared to PU-S_0 and confirming enhanced hydrophobicity. This enhancement arises primarily from the replacement of strongly polar -C=N- groups with weakly polar -S-S- moieties upon HEDS incorporation [3]. This substitution reduces the sample's surface energy and diminishes the hydrogen-bonding interactions between the original -C=N- groups and water molecules, thereby increasing the water contact angles of the PU-S series [4].

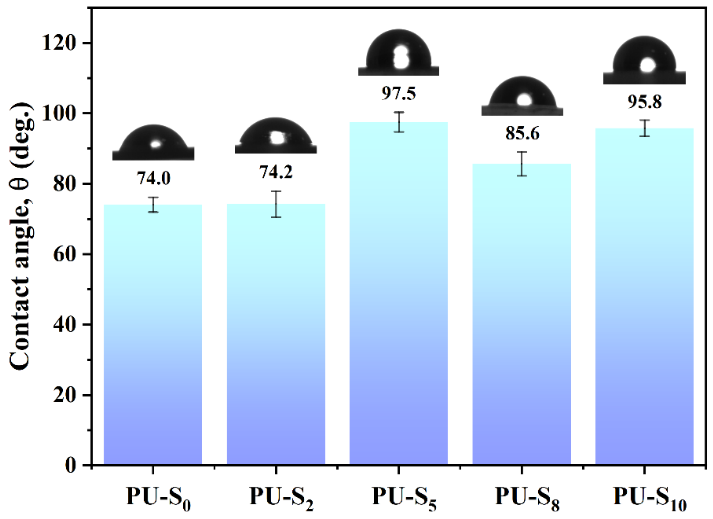

Figure S3. Water contact angle measurement of samples.

Figure S4 displays the XRD spectra of PU-S samples with different DMG and HEDS contents. In the XRD spectra, a broad diffraction peak was observed at 2θ = 20.3°, indicating that the molecular structure of the five samples showed an amorphous state and belonged to random polymers, which embodied the unique properties of PU. Analysis of the diffraction peak intensity variations in the PU-S samples reveals that with increasing HEDS content, the samples exhibit pronounced peak broadening and attenuation. This reduction in peak intensity reflects a decreasing trend in crystallinity for the PU-S series. Specifically, PU-S_0 displays sharp and intense diffraction peaks due to the strong polarity and hydrogen-bonding capability of the -C=N- groups. These intermolecular interactions promote ordered chain alignment and facilitate crystallization, leading to sharpened diffraction peaks [5]. However, the introduction of -S-S- disrupts the directional packing of hard segments and reduces hydrogen-bond density, consequently diminishing crystallinity and broadening the diffraction peaks.

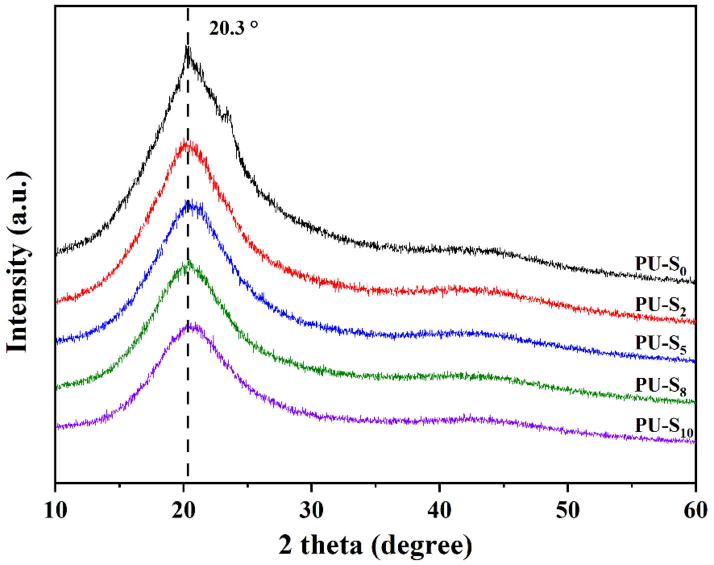

Figure S4. XRD spectra of samples.

The DSC curves of the PU-S films are shown in Figure S5, and the DSC data are summarized in Table S2. The glass transition temperatures (T_g) of PU-S_0, PU-S_2, PU-S_5, PU-S_8, and PU-S_{10} were observed at about –29.2°C, –29.6°C, –30.1°C, –30.5°C, and –31.3°C, respectively. The results show that the T_g of the samples gradually decreases with the increase of HEDS content. This reduction occurs because the incorporation of flexible -S-S- groups lowers the energy barrier for segmental motion. Concurrently, the decreased packing density of the flexible chains leads to an increase in free volume, thus causing the T_g to occur at a lower temperature [6].

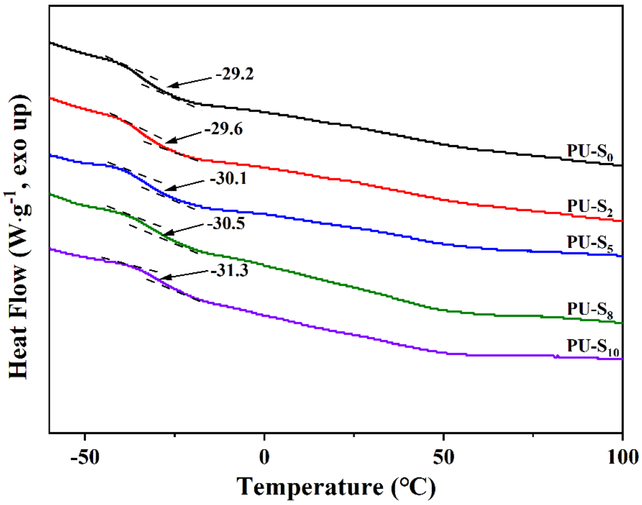

Figure S5. DSC curves of samples in the temperature range from –60 °C to 100 °C.

Table S2. DSC data of samples

Samples	Glass transition temperature (T_g, °C)
PU-S_0	–29.2
PU-S_2	–29.6
PU-S_5	–30.1
PU-S_8	–30.5
PU-S_{10}	–31.3

The dynamic mechanical properties of the samples with different DMG and HEDS contents were characterized by DMA, as shown in Figure S6. Additionally, it can be observed that as the HEDS content increases, the tan *δ* peak gradually shifts to a lower temperature position. Furthermore, the damping performance of the polymer shows a trend of first decreasing and then increasing with the HEDS content. Notably, PU-S_{10} with the highest HEDS content demonstrates higher damping characteristics over a broader temperature range. This is because the introduction of a small amount of -S-S- enhances the physical cross-linked network, resulting in the obstruction of molecular chain segment movement and a decrease in the local relaxation ability of the molecular chain, thereby reducing the viscous energy dissipation caused by chain segment friction. However, as the HEDS content increases, -S-S- forms a dense dynamic network, and the synchronous rupture and reformation of a large number of bonds under external force can significantly enhance the hysteretic energy dissipation. These observations align consistently with the aforementioned DSC test results [7].

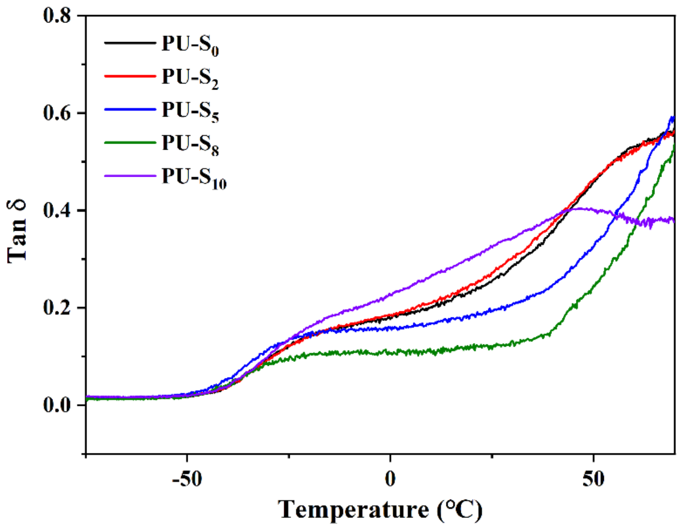

Figure S6. Tan δ curves of samples.

Figure S7 (a) shows the TG curves of the synthesized PU films, the corresponding temperatures at a weight loss of 5% ($T_{d,5}$) of PU-S_0, PU-S_2, PU-S_5, PU-S_8 and PU-S_{10} are 219.6 °C, 265.4 °C, 232.4 °C, 219.8 °C, and 274.2 °C, respectively (data summarized in Table S3). Moreover, as the DMG content decreases, the $T_{d,5}$ of PU-S progressively increases. This occurs because DMG contains C=N and N-O bonds in its structure. Such bonds, featuring electron-withdrawing atoms, exhibit relatively low cleavage temperatures. Consequently, DMG undergoes preferential decomposition, initiating chain scission that ultimately reduces the thermal stability of PU-S [8]. However, the temperature of the samples corresponding to a weight loss of 5% is above 210°C, and the components are still stable at room temperature without degradation, which can maintain the stability of the performance and ensure the normal use of the material [9].

Figure S7 (b) shows the DTG curves of the samples. As can be seen from Table S3, the temperature at maximum decomposition rate (T_{max}) of PU-S_0, PU-S_2, PU-S_5, PU-S_8, and PU-S_{10} are 339.9°C, 340.7°C, 341.1°C, 341.3°C, and 343.1°C, respectively. And among all the samples, PU-S_0 possesses the highest char yield at 700°C, which is 2.6%. The results show that PU-S_0, PU-S_2, PU-S_5, and PU-S_8 have three stages of decomposition, and the first stage belongs to the decomposition of the DMG. Additionally, the second stage is the decomposition of carbamate bonds, and the third

stage is the degradation of soft segments [10]. Furthermore, PU-S10 have two decomposition stages, the first one belongs to the hard stage, where the urethane bond starts to decompose, and the second one is the decomposition of the soft stage [11].

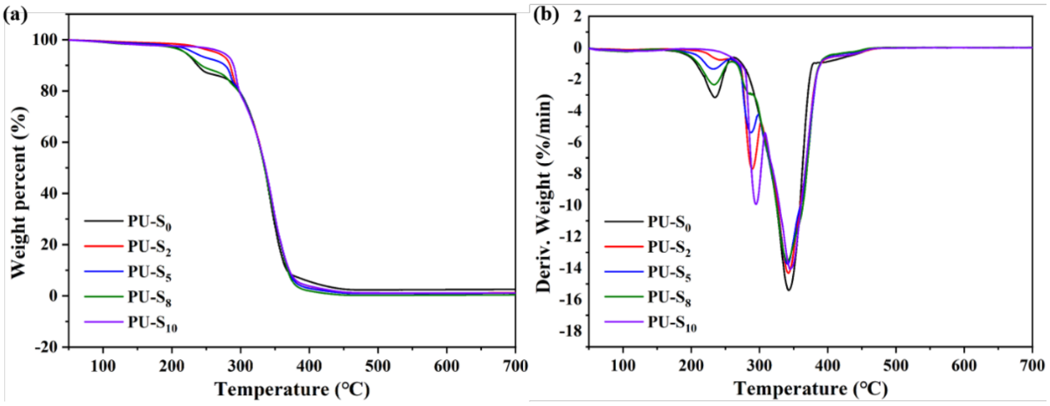

Figure S7. (**a**) TG curves of the samples; (**b**) DTG curves of the samples.

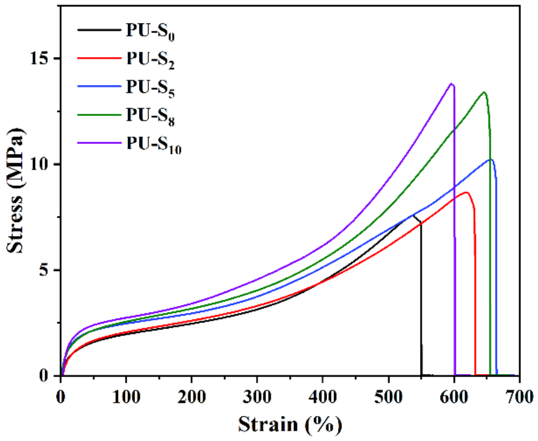

Figure S8. Stress-strain curves of the healed samples.

Table S3. The TGA data of the samples

Samples	$T_{d,5}$ (°C)	$T_{d,10}$ (°C)	T_{max} (°C)	$Y_{700°C}$ (%)
PU-S_0	219.6	237.9	339.9	2.6
PU-S_2	265.4	284.8	340.7	1.2
PU-S_5	232.4	277.4	341.1	1.1
PU-S_8	219.8	243.2	341.3	0.4
PU-S_{10}	274.2	289.8	343.1	1.1

$T_{d,5}$ refers to the temperature at 5% weight loss.

$T_{d,10}$ refers to the temperature at 10% weight loss.

T_{max} refers to the temperature at maximum decomposition rate.

$Y_{700℃}$ refers to char yield at 700 °C.

As shown in Table S4, both the tensile strength and elongation at break of the samples progressively increased with higher HEDS content. Specifically, PU-S_{10} exhibited the highest tensile strength (14.0 MPa), while PU-S_8 achieved the optimal elongation at break (656.2%).

Table S4. Mechanical characteristics of samples

Samples	breaking elongation (%)	$\sigma_{original}$ (MPa)	σ_{healed} (MPa)	η (%)
PU-S_0	586.6	11.2	7.6	67.9
PU-S_2	495.2	10.0	8.7	87.0
PU-S_5	558.6	11.3	10.2	90.3
PU-S_8	656.2	13.7	13.4	97.8
PU-S_{10}	611.0	14.0	13.8	98.6

breaking elongation refers to the tensile strength of original sample.

$\sigma_{original}$ refers to the tensile strength of original sample.

σ_{healed} refers to the tensile strength of healed sample.

η refers to the healing efficiency of sample.

References

1. Ghosh, T.; Karak, N. Biobased multifunctional macroglycol containing smart thermoplastic hyperbranched polyurethane elastomer with intrinsic self-healing attribute. *ACS Sustainable Chem. Eng*. **2018**, 6, 4370–4381.
2. Chen, T.; Fang, L.; Lu, C.; Xu, Z. Effects of blended reversible epoxy domains on structures and properties of self-healing/shape-memory thermoplastic polyurethane. *Macromol. Mater. Eng*. **2020**, 305, 1900578.
3. Jasmee, S.; Omar, G.; Masripan, N.; Kamarolzaman, A A.; Ashikin, A.; Ani, F C. Hydrophobicity performance of polyethylene terephthalate (PET) and thermoplastic polyurethane (TPU) with thermal effect. *Mater. Res. Express*. **2018**, 5, 096304.
4. Li, X.; Yu, R.; He, Y.; Zhang, Y.; Yang, X.; Zhao, X.; Huang, W. Self-healing polyurethane elastomers based on a disulfide bond by digital light processing 3D printing. *ACS Macro. Lett*. **2019**, 8, 1511–1516.
5. Zhang, Y.; Yu, Y.; Zhao, X.; Yang, X.; Yu, R.; Zhang, Y.; Huang, W. A High Strength but Fast Fracture-Self-Healing Thermoplastic Elastomer. *Macromol. Rapid Commun*. **2021**, 42, 2100135.
6. Xia, L.; Tu, H.; Zeng, W.; Yang, X.; Zhou, M.; Li, L.; Guo, X. A room-temperature self-healing elastomer with ultra-high strength and toughness fabricated via optimized hierarchical hydrogen-bonding interactions. *J. Mater. Chem. A* **2022**, 10, 4344–4354.
7. Fang, H.; Zhang, L.; Chen, A.; Wu, F. Improvement of mechanical property for PLA/TPU blend by adding PLA-TPU copolymers prepared via in situ ring-opening polymerization. *Polymers* **2022**, 14, 1530–1541.
8. Wang, X.; Zhang, H.; Yang, B.; Wang, L.; Sun, H. A colorless, transparent and self-healing polyurethane elastomer modulated by dynamic disulfide and hydrogen bonds. *New J. Chem*. **2020**, 44, 5746–5754.
9. Ma, H.; Fashandi, M.; Rejeb, Z B.; Ming, X.; Liu, Y.; Gong, P.; Li, G.; Park, C.B. Efficient electromagnetic wave absorption and thermal infrared stealth in PVTMS@ MWCNT nano-aerogel via abundant nano-sized cavities and attenuation interfaces. *Nano-Micro. Lett*. **2024**, 16, 20–33.
10. Yuan, B.; Ritzoulis, C.; Chen, J. Extensional and shear rheology of a food hydrocolloid. *Food Hydrocolloids* **2018**, 74, 296–306.
11. Bi, H.; Ren, Z.; Ye, G.; Sun, H.; Guo, R.; Jia, X.; Xu, M. Fabrication of cellulose nanocrystal reinforced thermoplastic polyurethane/polycaprolactone blends for three-dimension printing self-healing nanocomposites. *Cellulose* **2020**, 27, 8011–8026.
